# Perspectives of nursing students on challenges of e-learning during early stages of the COVID-19 pandemic

**DOI:** 10.4102/curationis.v46i1.2358

**Published:** 2023-01-26

**Authors:** Vistolina Nuuyoma, Sydney S. Lauliso, Leonard Chihururu

**Affiliations:** 1 School of Nursing and Public Health, Faculty of Health Sciences and Veterinary Medicine, University of Namibia, Rundu, Namibia

**Keywords:** e-learning, e-learning challenges, COVID-19 pandemic, online learning, nursing education, resource-constrained settings, web-based learning

## Abstract

**Background:**

E-learning is becoming an important approach to teaching and learning in higher education institutions, including nursing training. Despite that, there are students who were never introduced to e-learning prior to the coronavirus disease 2019 (COVID-19) pandemic. Their challenges in relation to e-learning could differ from those of other students who had experienced the platform before, especially against the backdrop of the COVID-19 pandemic that brought an abrupt change in the approach to teaching, learning and assessment.

**Objectives:**

This study explored and described university nursing students’ challenges in relation to e-learning during the early stages of the COVID-19 pandemic in a resource-constrained setting.

**Method:**

Qualitative exploratory and contextual design was used. The sample consisted of 17 participants who were conveniently selected, and data were collected by means of two focus groups and five individual interviews. Data analysis followed a qualitative content analysis process.

**Results:**

The five categories emanated from analysis are e-learning mode not suitable for practical components, challenges related to assessment of learning, connectivity issues, e-learning is a lonely journey and computer illiteracy and limited skills for the use of e-learning.

**Conclusion:**

Nursing students’ challenges regarding e-learning during the early stages of the COVID-19 pandemic related to the learning of practical components, assessment, connectivity, a lack of interaction with peers and a lack of the skills required to operate e-learning tools.

**Contribution:**

The findings have implications for international, regional and local contexts in helping to develop support systems and preparing students to use e-learning when it is introduced abruptly.

## Introduction

E-learning is an innovative web-based system founded on digital technologies and other forms of educational materials, the principal goal of which is to offer students an open, learner-centred, personalised, enjoyable, supportive and interactive learning environment that enhances the learning processes (Rodrigues et al. 2019:95). E-learning stands for electronic learning, which is an alternative to traditional education and may be complementary to it (Basak, Wotto & Bélanger 2018:191). According to Abed (2019:1), e-learning is an umbrella concept referring to the delivery of educational content to recipients via media, and its networks in such a way that an opportunity is allowed for active interaction with the content. The type of tools used for e-learning should contain features that allow for synchronous or asynchronous interaction between learners and facilitator as well as learners and peers. In addition, there should be the possibility of completing learning activities within the time allocated by the facilitator and at a speed that suits the conditions and the learning outcomes of the course (Abed 2019:1). In a Delphi study conducted by Sangrà, Vlachopoulos and Cabrera (2012:152), e-learning is defined as an approach to teaching and learning that is built on the use of electronic media and devices as tools for expanding access to training, interaction and communication, as well as facilitating the adoption of new ways of conceptualising and developing learning.

The four fundamental perspectives of e-learning are interdependent and comprise cognitive, emotional, behavioural and contextual perspectives (Basak et al. 2018:197). From a cognitive perspective, the tools used in e-learning should teach learners social, communication and collaborative skills to assist them to form dialogue, interact and experience vicarious learning (Tlambda 2014:n.p.). Emotional perspectives on e-learning place an emphasis on engagement and the emotional aspects of learning. For behavioural perspectives, e-learning tools should be able to assist learners to achieve behavioural outcomes of learning that may be role-modelled and applied to clinical settings, while the contextual perspectives focus on environmental and social aspects that may stimulate learning.

Other concepts applied to e-learning include virtual learning, online learning, distributed learning and network and web-based learning (Chitra & Raj 2018:11). E-learning is becoming an important approach to teaching and learning in higher education institutions, including in nursing curricula (Ali 2016:2). Accordingly, e-learning forms part of the new dynamic that symbolises educational systems at the beginning of the 21st century (Sangrà et al. 2012:145). E-learning has been found to be a convenient approach to education because it enables students to fit learning into their lifestyles, allowing even a person with the busiest schedule to pursue a career and obtain new qualifications successfully (Chitra & Raj 2018:12). This is because students studying through e-learning can access the course content at any time and use the resources as desired. Furthermore, e-learning allows students to use a diversity of learning styles (Caliskan, Tugun & Uzunboylu 2017:40). Other potential benefits of e-learning for students include access to learning tools and resources such as online discussions, email, video and evaluations. These make e-learning a useful tool for enhancing the quality of teaching and learning (Ali 2016:3).

Generally, information and communications technology (ICT) has been used in nursing education for various reasons such as searching for information and learning resources for class activities, the assessment of learning, self-directed learning and the professional development of educators, creating digital materials for students and uploading homework for them on school websites (Harerimana & Mtshali 2017:26). E-learning was used in both undergraduate and nursing programmes prior to the coronavirus disease 2019 (COVID-19) pandemic. It may be introduced as part of blended learning in a face-to-face course in an undergraduate programme or fully online courses for distance courses, especially in postgraduate nursing training. E-learning models applied in nursing education are designed with consideration of components such as student needs, infrastructure, support systems, educational aspects such as teaching, learning and assessment, adherence to ethics law related to ICT, related professions and institutions, cultural aspects pertaining to facilitators, students and society at large and evaluations of content, facilitators and systems (Raoufi et al. 2020:13).

An e-learning approach differs from a face-to-face approach, and therefore the experiences of both students and educators also differ. Hence, for students to benefit from an e-learning approach, they should possess online learning readiness (Engin 2017:36). Online learning readiness encompasses the possession of technical, learning and time management skills, as well as internet connection; these factors all contribute to the learning experiences of learners, as well as educators’ experiences.

Owing to the COVID-19 pandemic, educational institutions worldwide shut down to curb the spread of the virus. Thus, globally, the immediate response of educational institutions was to resort to e-learning as a means to continue education during the pandemic (Cuisia-Villanueva 2020:1). Apart from the continuation of education during the early stages of the pandemic, the sudden transition of teaching and learning activities onto e-learning platforms was carried out to avoid mass gatherings, which were potential risks for the spread of the virus (Amir et al. 2020:2). Worldwide, COVID-19 pandemic protocols dictated that students and educators should work remotely by implementing full distance learning. Various online platforms such as Google Meets, Microsoft Teams, Zoom and Moodle-based learning management systems were utilised. According to Amir et al. (2020:3), practical modules and courses with learning outcomes involving psychomotor skills, which usually require skilled laboratory practice, were postponed until face-to-face interactions were permitted or in some cases substituted with live online presentations or recorded simulation sessions. Namibia reported the first case of COVID-19 in March 2020; as a result, one public university resorted to the use of Moodle as a learning management system for all programmes, including health science programmes such as nursing. In addition, other online tools used to facilitate teaching and learning during pandemic lockdowns in Namibia included WhatsApp, Telegram, Google Meet, Microsoft Teams, Zoom and emailing.

E-learning is growing in educational systems throughout Africa, and challenges were faced by students and educators even before the COVID-19 pandemic. Challenges related to e-learning identified in Botswana were poor infrastructure such as poor internet services, inadequate computer laboratories and constant power outages. Other challenges involved a lack of policy on e-learning, inadequate information technology (IT) support and a lack of university management support (Moakofhi et al. 2017:10). In addition, some instructors facilitating courses via the e-learning mode are unskilled in the use of technology (Ngampornchai & Adams 2016:7). Interestingly, Palvia et al. (2018:237) stress that it is important that the harmful effects of technology such as addiction to smartphones are also taken into consideration, especially during the formulation of e-learning policies for better educational outcomes. This may be because addiction to the use of smartphones could be a potential problem when using e-learning, especially in the developing world. A study conducted in Namibia revealed e-learning challenges to include a lack of access to devices, the slow speed of internet connections and lack of and limited access to internet connectivity (Mässing & Söderström 2017:28). The studies discussed here regarding the challenges of e-learning were conducted prior to the COVID-19 pandemic and mostly on distance students who were prepared for this mode of learning. Therefore, students’ experiences and challenges may differ when e-learning is abruptly introduced, especially among students studying via a full-time, face-to-face mode of delivery, as was the case in the early stages of the COVID-19 pandemic.

The COVID-19 pandemic had a negative impact on nursing education. As a result, groups of nursing students who were studying during the pandemic encountered unparalleled difficulties, having obligations to meet the academic requirements of their programme while taking on extra clinical commitments and trying to keep themselves and their families safe from contracting the virus (Barrett 2022:38). Owing to the pandemic conditions globally, the motivation of nursing students decreased, leading to their being unable to master knowledge and skills, delayed periods of study for some that led to the extension of training and ultimately increased stress levels (Zendrato & Hiko 2021:582).

A study conducted in Turkey revealed that challenges for nursing students related to the use of e-learning during the COVID-19 pandemic included psychological difficulties, uncertainties, insufficiencies regarding education, restrictions concerning social life and family conflicts (Cengiz, Gurdap & Işik 2022:49). In addition, a narrative review by Divya and Binil (2021:2319) indicated challenges related to the use of e-learning during the pandemic such as the fact that students were missing out on the real essence of practical aspects of nursing care, international training was cancelled, and students experienced the loss of job opportunities owing to a lack of confidence in their skill. Additional challenges experienced by nursing students during the pandemic included internet connectivity issues, problems with electricity and lack of computer literacy.

A study conducted in Iran revealed inappropriate groundwork such as a lack of facilities and equipment, economic hardship and lecturers’ lack of preparedness for the virtual classroom. In addition, a low inclination for virtual education such as low students and lecturers adherence to e-learning was revealed as challenges of the sudden shift to asynchronous virtual education in nursing training during the pandemic (Moradi 2022:46). A study carried out in the United Arab Emirates on nursing students’ challenges relating to e-learning encountered during the COVID-19 pandemic included lack of robust internet coverage affecting students’ engagement on learning activities, concerns about lack of privacy, difficulties with the teaching platforms, internet connection issues and communication difficulties. In addition, there were concerns that facilitators were slow at reacting to their needs (Mukasa et al. 2021:1508).

According to James (2021:5), students’ performance in e-learning depends on the interplay of factors, ranging from learners’ motivation, behaviours, learning styles and level of computer skills. In addition, factors such as institutional or administrative support, environmental factors, system configuration and technical design, as well as instructors’ characteristics, determine student performance. Therefore, it is necessary for higher education institutions to understand the challenges and experiences of students in relation to e-learning so as to assist in the implementation and redesign of the support systems needed for successful educational processes and performances. An understanding of the challenges nursing students experienced regarding e-learning during the early stages of COVID-19 is needed for institutional preparations in the case of future pandemics. This may contribute to the development of student learning supportive systems during times of uncertainties, as in the case of pandemics, to eliminate inefficiencies and produce well-trained and competent nurses.

Although e-learning has been actively used in higher education for the past two decades, there are students who had never been introduced to e-learning prior to the COVID-19 pandemic. This includes the 2020 final-year nursing students at a public university in Namibia, who had been taught all courses via a face-to-face mode since beginning of their training in 2017 (Nuuyoma & Chihururu 2021). Their experiences, perceptions and challenges relating to e-learning were not known as no prior assessment and exploration had been done in the study setting. Their experiences and challenges of e-learning could differ from those of other students who had experienced the platform before and, also considering this was an abrupt change in teaching, of the learning and assessment approach. Therefore, this study was conducted to explore and describe university nursing students’ challenges of e-learning during early stages of the COVID-19 pandemic in a resource-constrained setting.

## Research design and methods

### Research design

The study followed a qualitative exploratory and contextual design as described by Polit and Beck (2017:52). An exploratory design has the advantage of discovering the full nature of a phenomenon in terms of what is really going on and how the phenomenon is experienced, while a contextual design has the advantage of discovering a contextualised understanding of human experiences (Polit & Beck 2017:52 & 881). Therefore, a qualitative exploratory and contextual design allowed for an in-depth exploration and understanding of the e-learning-related challenges university nursing students faced during the COVID-19 pandemic within their study context.

### Study setting

The study setting is a campus of a public university located in one of the north-eastern regions of Namibia. The university campus offers an academic programme in the fields of nursing, education, economics and management sciences. The nursing qualifications offered on campus include a 4-year, full-time undergraduate Bachelor of Nursing Science (clinical) Honours programme. Prior to the COVID-19 pandemic, all nursing courses in this programme were offered via a face-to-face mode.

### Population and sampling

The targeted population for this study was fourth-year nursing students. This population group was targeted because it had never been introduced to e-learning prior to the pandemic. By contrast, the other groups of nursing students in their first- to third-year levels of training had been exposed to e-learning prior to the pandemic with some courses such as English, contemporary social issues and online computer literacy being offered. Meanwhile for students in their fourth year, these were offered via a face-to-face mode. A total of 86 students were registered at the fourth-year level at the time of data collection. Accordingly, participants were conveniently selected. Convenience sampling involves selecting readily available participants. This was seen as a suitable sampling technique for this study as the intention of qualitative research is not to generalise findings (Brink, Van der Walt & Van Rensburg 2018:125). The other reason for choosing convenience sampling is that all 2020 final-year nursing students at a public university in Namibia were taught all courses via face-to-face mode since beginning of their training and thus it was considered a suitable technique to select participants. The prospective participants were fourth-year nursing students who were accessible, available during data collection period, willing to participate in the study and have given written informed consent. Fourth-year nursing students who were not accessible or available during data collection period and not willing to participate in the study were excluded. The research sample consisted of 17 participants, and this was determined by data saturation. Therefore a sample of 17 participants was considered rich and adequate as no new insight emerged and responses started to yield repetitive information (Brink et al. 2018:160). In addition, Lowe et al. (2018:200) indicate that data saturation occurs when maximal information has been obtained from research participants; in the current study this occurred with 17 participants.

### Data collection

Data were collected from 01 June 2020 to 25 July 2020 using two focus group interviews with six participants each and five individual interviews. Initially, the researchers planned to collect data via focus group interviews only; however, owing to an increase in COVID-19 cases, it was not possible to get participants together; therefore some were interviewed individually. Participants were approached via a message sent to the fourth-year nursing students WhatsApp group and instant messages sent to the cell phone numbers of all the students in this cohort. Participants who were available and interested in the study made arrangements with the researcher who collected the data with regard to times and dates for the interviews. Arrangements were made to ensure that data collection did not interfere with learning activities.

In both the focus group and the individual interviews, qualitative tool used during data collection are topic guide, digital voice recorder, pen and notebook for field notes. A topic guide was used to facilitate data collection. The topic guide consisted of a central question: ‘Tell me about your challenges with the use of e-learning as a nursing student during the early stages of the COVID-19 pandemic’. This was followed by probes and several prompts from the interviewer in order to help obtain a deeper understanding of participants’ responses and also to create a dialogue (Moser & Korstjens 2018:13). The topic guide was developed by both researchers and was piloted with two participants who were interviewed individually prior to the main data collection process. To create a dialogue during data collection, the researcher used facilitative communication techniques such as listening, probing, summarising, paraphrasing and reflecting.

The focus group interviews were mediated by one researcher, who also conducted the individual interviews. The focus group interviews took place in the classrooms at the university campus, while individual interviews took place at the participants’ residences and at the campus. No one apart from the research participants attended the focus group interviews or listened in on the individual interviews. This ensured confidentiality during data collection. All interviews were conducted in English, and audio recordings were made using a digital voice recorder to help retain the responses needed for data analysis. Permission for recording the interviews was obtained at the same time that consent for participation in the research was sought. All audio recordings were transferred for storage in a computer protected with a password and only the research team listened to them – this further ensured confidentiality. All audio recordings were transcribed verbatim and were returned to participants for member checking (Brink et al. 2018:111). The researcher who collected the data also made use of field notes to record all nonverbal clues, group interactions, personal reflections and all other aspects noted during the data collection process that were not possible to include in the audio recordings. Individual interviews lasted for 37–40 min, while focus group interviews ranged between 47 min and 56 min.

### Data analysis

All audio recordings were transcribed verbatim, and data were analysed manually according to the Bengtsson (2016:9) process of qualitative content analysis. This process consisted of the following stages: decontextualisation – in this stage, meaning units were identified and the researchers created a code list. In stage two, recontextualisation, the original text was reread alongside the final list of meaning units and field notes made during data collection. In stage three, categorisation was done by condensing meaning units and allocating them to domains. These domains were identified as categories. During the last phase, compilation, the researchers drew conclusions regarding the final categories of results, which also involved peer debriefing by researchers who were not part of this study and member checking by study participants. The data analysis of each transcript was conducted individually by both researchers who then met to agree on the final categories of results. Two of the researchers are experienced in conducting qualitative research and were also engaged in peer debriefing during the research process to ensure trustworthiness.

### Trustworthiness

Study rigour was ensured based on the four criteria for developing trustworthiness in qualitative research. These criteria are credibility, dependability, confirmability and transferability (Lincoln & Guba 1985:290–331). Credibility was maximised by the use of audio recordings during the data collection process; the collection of data until saturation was reached, peer debriefing, member checking, the reflexivity by researchers and prolonged engagement. Dependability was ensured via stepwise replication during data analysis and peer debriefing. Reflexivity, triangulation of the researchers during data analysis and including direct quotes from participants in the presentation of findings were measures used to ensure confirmability. The collection of data until saturation was reached and thick descriptions through the provision of detailed descriptions of the data within the research context and their reportage were the measures incorporated in this study to help readers make judgements regarding transferability.

### Ethical considerations

Ethical clearance to conduct this study was obtained from the University of Namibia School of Nursing Research Ethics Committee (No. SoNEC VN1/2020). Permission to conduct the study was also granted by the head of the school. Moreover, participation in the study was voluntary, and participants gave written informed consent prior to participation in the study. This was done by signing consent forms to agree to participate and for recording the interviews. The confidentiality and anonymity of the participants was ensured, as their names were not mentioned during data collection, analysis and report writing; instead a number was allocated to each participant. Nonmaleficence was ensured by only collecting data that were needed to reach the objectives of the study and participants were assured that information obtained would only be used for research purposes. Beneficence was guaranteed by ensuring that participants were not exposed to any form of risk or harm as a result of participating in the study. Justice was ensured through fair and equal treatment of all participants.

## Results and discussion

### Demographic characteristics of participants

Of the 17 participants, 10 were females while 7 were male. Their age ranges were 21 to 29 years, and all participants indicated single as their marital status during the data collection period. All participants were registered for their final year in the Bachelor of Nursing Science (clinical) Honours programme, and none had a qualification in IT or related fields. Only four participants were residing in the university hostel during the data collection period and the rest were either staying in rented accommodation outside the campus or living with family members.

**FIGURE 1:**
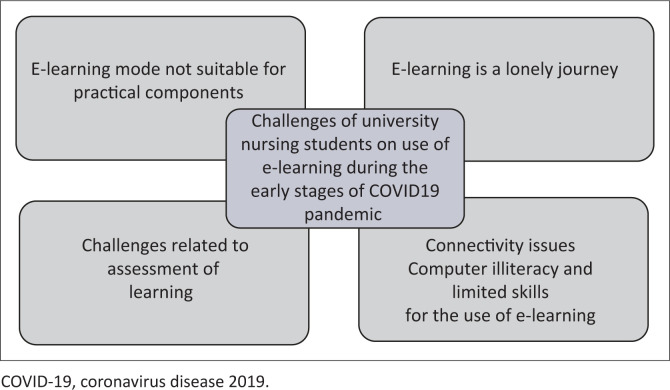
Summary of study results.

Five categories emanated from the qualitative content analysis: e-learning mode not suitable for practical components, challenges related to the assessment of learning, connectivity issues, e-learning is a lonely journey and computer illiteracy and limited skills for the use of e-learning. A summary of the study results is displayed in Figure 1 and Table 1.

**TABLE 1:** Categories and subcategories as results of the study.

Categories	Subcategories
1. E-learning mode not suitable for practical components	1.1. No practical learning via e-learning 1.2. Students behind with practical work because of use of e-learning
2. Challenges related to assessment of learning	2.1. A lot of time is required for online assessment 2.2. Unable to operate a device and think at the same time 2.3. No supervision during online assessment 2.4. Students copy from others
3. Connectivity issues	3.1. Poor internet connectivity 3.2. No adequate internet data 3.3. Useful websites not accessible during the day
4. E-learning is a lonely journey	4.1. No peers to assist 4.2. No group works and interactions 4.3. Students stayed in isolation 4.4. Students relocated to places with no friends and peers
5. Computer illiterate and limited skills on the use of e-learning	5.1. No orientation on management learning system 5.2. Limited skills to operate e-learning tools 5.3. Learning management system not user-friendly 5.4. Computer illiterate

### Category 1: E-learning mode not suitable for practical components

Health science education includes practical sessions in anatomy, physiology and biochemistry held at clinical skills centres, coupled with bedside training that entails the physical presence of educators and peers (Olum et al. 2020:5). In addition, nursing training is practice based, which is emphasised through the clinical placement of students to integrate theoretical knowledge with the real world of clinical practice (Alshahrani, Cusack & Rasmussen 2018:104). The participants in the current study stated that e-learning is not conducive for learning, teaching and assessing practical components of nursing as they are required to demonstrate clinical competencies. This was mentioned by participants:

‘On my side as a nursing student, e-learning is very challenging because it is not possible to do practical online, I mean like not possible to do clinical procedures online. We nursing students are expected to do this … I think e-learning is only good and beneficial to other courses that don’t need practical work.’ (Participant 4, Focus Group Interview [FGI] 1, 29-year-old male)

‘With the use of e-learning, I can say there is nothing we are doing regarding clinical component, our lecturers always tell us, we will do that when you are allowed to come to simulation and clinical training facilities, it’s like e-leaning is only for theory.’ (Participant 2, Individual Interview [II], 21-year-old female)

This finding concurs with Puljak et al. (2020:7) who conducted a study on the attitudes and concerns of undergraduate university health science students regarding a complete switch to e-learning during the COVID-19 pandemic in Croatia. They also revealed concerns regarding the lack of practical education during the use of e-learning. Moreover, their results revealed students were concerned that the lack of practical education might have enduring consequences for their job preparedness. The lack of practical and clinical exposure during the use of e-learning in health science students was also highlighted as a major challenge in Uganda (Olum et al. 2020:5). Similarly, health science students in Saudi Arabia indicated that clinical teaching is not suitable for e-learning platforms and that they faced many challenges in achieving practical learning outcomes as a result of this (Ibrahim et al. 2021:20). Suspension of practical and clinical training of health science students, in which nursing is included, may also have contributed to students’ disagreement on the quality of their study and their concerns regarding their clinical skills acquisition, leading to high level of anxieties (Almoayad et al. 2020:322).

Nursing students in the current study experienced anxiety owing to the unsuitability of e-learning for learning practical components and also because of the resulting lack of practical opportunities. Owing to the pandemic lockdowns, all health science students’ clinical placements at a public university in Namibia were put on hold and therefore no teaching and learning took place, except for the theoretical components that were conducted through e-learning. This was mentioned as follows by one of the participants:

‘We are already behind with practical works, like we were supposed to be allocated in surgical ward but the whole two months we were not allowed to go to the hospital. The main problem is that we are not able to cover clinical or practical part via e-learning.’ (Participant 5, FGI2, 28-year-old female)

‘My worry is more about becoming a competent nurse, will this e-learning thing really make us ready for the job market?’ (Participant 6, FGI1, 23-year-old male)

While nursing students in the current study were restricted from attending clinical practice where they learn clinical components of nursing, nursing students from other settings were allowed to go for clinical attachment during the use of e-learning amid COVID-19 pandemic. To support this, Barrett (2022:39) reported that the pandemic gave nursing students the chance to gain practical hours, practise their skills and contribute directly to the care of those suffering from COVID-19.

### Category 2: E-learning challenges related to the assessment of learning

Participants revealed that e-learning does not seem suitable for assessment. This is because in most cases, time allowed for assessment is not adequate for students to respond to all questions. For example, the online test may include structured questions that require thought and the typing of answers, which participants indicated needed a lot of time and concentration. In some cases, it led to students not completing assessments and thus performing poorly. Participants also indicated that they find it hard to think, operate the device keyboard and focus on the screen at the same time:

‘I find it difficult to do assessment online, especially structured questions. I think I am yet to master the skills of typing and think at the same time. Sometimes we do not finish the test because structured questions require a lot of time.’ (Participant 2, II, 23-year-old female)

‘It was challenging really when it comes to writing tests online, me personally, on 3 occasions, I don’t manage to complete tests because it’s very different when you write test on paper and a pen, you know … I have to think, back to the key board of my laptop, back to the screen, it just didn’t work for me …’ (Participant 3 FGI1, 24-year-old male)

While a previous study associated challenges related to assessment in e-learning modes during COVID-19 as internet access, which negatively affected student performances (Adarkwah 2020:10), the current study associated assessment challenges with the structure of questions, the duration of online tests and focusing on operating devices and cognitive application. The structured questions did not seem to match the time allocated for online tests. However, a study conducted in Jordan revealed that students showed a high acceptance of online assessment during the COVID-19 pandemic because it serves as a flexible assessment method (Alsalhi et al. 2022:41). In another study conducted in the Philippines, students liked being assessed online because they received immediate feedback, which is a motivating factor for learning (Valdez & Maderal 2021:422).

Furthermore, participants in the current study reported that students copied from each other during online tests. Although the university does not allow such practices and they are punishable when evidence is produced, it is difficult to find evidence for cheating as online tests are conducted without supervision. One participant had the following to say:

‘E-learning itself got a challenge like when it comes to assessment since students are not supervised, they cheat by copying from their friends and no one will know. Seriously, there are students who copy from friends every time they write a test, what are you learning? you are not learning anything because you are just copying or you just tell your friend to do for you the test because they studied, then you just pass the test, so at the end of the day you are not learning anything.’ (Participant 4, II, 23-year-old male)

Concerns for students’ dishonesty during online assessment, specifically amid the COVID-19 pandemic, were also raised in a previous study conducted on health science students in Indonesia (Amir et al. 2020:6). However, these concerns over dishonesty in the use of e-learning were also raised prior to the COVID-19 pandemic. In a study conducted in Botswana, educators revealed concerns that academic dishonesty might increase with the introduction of e-learning. This was associated with concerns that the system might allow unauthorised individuals to access learning material and online tests and that students might be involved in plagiarism (Moakofhi et al. 2017:11).

### Category 3: E-learning is a lonely journey

During normal university operations, nursing students meet with other students on the campus where they attend classes, participate in learning activities in the library such as using computers, socialise with their peers and share learning materials. However, there was an abrupt change during early stages of the pandemic. Students were not permitted to enter the campus, therefore being expected to change their routine as well as make alternative plans for accessing e-learning. In the process, some had to spend longer periods without seeing their peers owing to their relocation to other towns and regions. As students were used to peer interactions and learning as a group, it was challenging for them to cope with e-learning as they had to do most activities individually. This was mentioned by the participants as follows:

‘The challenge with e-learning is that, I have to do everything by myself. I am not used to that, I always ask my classmates in class or when we are in clinical settings.’ (Participant 1, II, 22-year-old female)

‘It was difficult for me because I have to go back home, my place is 450 km from here. Every time I am studying, I think of my classmates, I had no one to ask or discuss with. It was not possible to call because calling requires money and time, which I didn’t have.’ (Participant 5, II, 23-year-old male)

‘E-learning during early days of pandemic was challenging, like for me, I stay alone and we were not allowed to move around, I felt isolated from my peers, I was craving that normal interaction we always had with my group member.’ (Participant 5, FGI2, 28-year-old female)

The findings on isolation and the eagerness for group learning reflected by the participants in this study were not surprising. Students’ concerns relating to interactions with each other during e-learning have also been reported in previous research (Adarkwah 2020:8; Gismalla et al., 2021:6).

Although e-learning during the early stages of COVID-19 was viewed as a lonely journey in the context of the current study, a study conducted in India revealed that the majority of the students opted for online classes during the pandemic. This is because they found it to be more flexible and convenient, which makes learning attractive (Muthuprasad et al. 2021:4). In another study, when participants were asked about their learning mode preferences for the next academic year, the majority of medical students in Saudi Arabia preferred online learning (Khalil et al. 2020:6).

### Category 4: Connectivity issues

E-learning platforms depend greatly on the internet; therefore both educators and students are required to have a good internet network (Minghat et al. 2020:18). Previous studies conducted on the use of e-learning during the early stages of the COVID-19 pandemic revealed lack of access to good quality internet, power failure and unstable internet connection as some of the challenges experienced (Adarkwah 2020:10; Amir et al. 2020:3; Divya & Binil 2021:2319; Mukasa et al. 2021:1508). Poor internet signals in some parts of the world are because of geographical limitations, which educational institutions have no control over (Gupta et al. 2020:22). In the current study, poor and lack of access to internet hampers e-learning as students find it difficult to attend live sessions, as well as downloading learning materials. In some cases, it led to an extra financial burden as they strived to purchase extra internet quotas for better connectivity.

In the current study setting, university students receive sim cards for mobile internet devices, which are distributed by the university in partnership with a local telecommunications company. Their sim cards are allocated an internet quota each month, and students are expected to top up when the allocated quota is used up. However, this does not address the challenges related to internet connectivity because during lockdown periods, as university hostels closed, most students resided in remote areas where they did not have internet reception. In addition, in the part of the country where the current study was conducted, internet connection through mobile internet devices is generally slow during the day, forcing students to do learning activities late at night or early in the morning. As an alternative, students were expected to purchase data from a different supplier to use on their smartphones or tablets. This is because this supplier has better internet coverage in almost all parts of the country than the provider that is connected to their university-provided sim cards. This was mentioned as follows:

‘The challenges that I have experienced with e-learning is trouble with the network connectivity, sometimes you find yourself in an area where network is very poor and you can’t access e-learning, like you fail to do activities on time, like tests and its trouble for you.’ (Participant 2, FGI1, 26-year-old female)

‘The first challenge with e-learning I would say, it’s expensive like for us we’re given TN mobile cards, like some phones only have 3G network, no 4G but then with those cards if you use 3G network it will take 2 minutes to load a page, so it’s very slow unless you have 4G, so if you have a phone which does not support 4G, it means you will be forced to buy a phone which supports a 4G sim card which is expensive.’ (Participant 6, FGI2, 22-year-old male)

### Category 5: Computer illiteracy and limited skills for the use of e-learning

For e-learning to have a positive impact and to be a suitable alternative learning mode, good preparation is needed for students and lecturers before activities can be effectively implemented (Minghat et al. 2020:22). Participants in the current study indicated that they struggled with e-learning because it was used with this group for the first time during the early stages of the COVID-19 pandemic. The struggle with e-learning was associated with a lack of and limited computer skills. Despite the fact that the university offers computer literacy as a core course to all first-year students, it seems students did not accumulate adequate skills and knowledge to help them navigate e-learning platforms. Participants also alluded to the fact that when e-learning was abruptly introduced during the lockdowns, the students received no preparation or orientation on how to navigate the learning management system. In addition, participants indicated that some educators appeared to have limited skills for the use of e-learning, finding it challenging to deliver the content and conduct assessment. This was mentioned as follows by participants:

‘We were not prepared to use e-learning, that’s why I find it difficult to navigate through the system because I have limited knowledge and skills of how computers work.’ (Participant 6, II, 21-year-old female)

‘I think the other problem also is, the lecturers are really not good at using this e-learning thing, so I think that lecturers also need to be educated on how to use these technological platforms because they end up making mistakes like maybe the way they are setting up questions for activities then we end up making mistakes and all that, if they do not know how, they find it difficult to teach and do assessment.’ (Participant 3, II, 23-year-old female)

‘Sometimes also it’s difficult to get feedback, like some of us did not do computer in school, so computer literacy comes in, growing up was a bit challenging and getting to know computer at a late age when you are in adulthood its somehow also a challenge.’ (Participant 4, FGI1, 23-year-old female)

Although e-learning was found to be the best solution for an online interactive learning environment for health science students during the COVID-19 pandemic (Gismalla et al., 2021:6), it may have been hampered by issues such as a lack of adequate computer skills and the unavailability of adequate training in the area of information and technology (Ibrahim et al. 2021:21). Moreover, unfamiliarity with e-learning platforms was previously reported as a challenge of e-learning for both educators and students. The findings of the current study on the unpreparedness and lack of orientation to e-learning platforms before implementation concur with Adarkwah (2020:10), who indicated that ICT tools were not given prior to e-learning and students had little exposure to online learning.

The findings of the current study have implications for local, regional and international contexts. For local context, there are many new nursing training institutions with students who were not exposed to e-learning prior to COVID-19 pandemic because the programmes are delivered in a full-time, face-to-face mode. Hence, the findings of the current study exposed the challenges of e-learning as experienced by students who had never been exposed to e-learning. This may assist in selecting suitable tools for e-learning implementation and also in seeking assistance from stakeholders to mitigate the challenges experienced. Within regional and international contexts, the study findings contribute to the body of knowledge available on e-learning challenges. This makes a unique contribution as the study was conducted in a resource-constrained setting, during the early stages of the COVID-19 pandemic, thus giving lessons for preparation in the case of future pandemics that may also require abrupt shifts in learning modes.

## Conclusion

E-learning provided the best alternative and immediate response for educational institutions to continue with teaching and learning during the COVID-19 pandemic, at the same time curbing the spread of the virus by avoiding large gatherings and social interactions. However, e-learning may be challenging, especially in resource-constrained settings and when introduced abruptly to students for the first time. Therefore, this study was conducted to explore and describe university nursing students’ challenges in regard to e-learning during the early stages of the COVID-19 pandemic in a resource-constrained setting. The findings revealed that an e-learning mode is not suitable for practical components, issues related to the assessment of learning, connectivity issues, the fact that e-learning is a lonely journey, as well as computer illiteracy and limited skills for the use of e-learning as e-learning–related challenges during the early stages of the COVID-19 pandemic. These findings have implications for local, regional and international contexts.

### Limitations of the study

The study was conducted on a population from a rural campus, located in a resource-constrained setting. Therefore, generalisations to students in cities and urban-based campuses may not be possible as challenges may differ as a result of the infrastructure and services available in different geographical locations. Another limitation is that participants were recruited via the WhatsApp platform and instant messages were sent to students; although data saturation was reached, there might have been some students who were willing to participate but did not have internet data or airtime to contact the researchers. Future researchers could explore the challenges of nursing students who have previously been exposed to e-learning, as the population in the current study setting had never been exposed to online learning prior to the COVID-19 pandemic lockdowns.

### Recommendations

Based on the findings of the current study, it is recommended that nursing educators find alternatives so that students can continue practical learning during the use of e-learning. This may be done by incorporating problem-based learning, case studies, forum discussions, virtual classrooms and the use of teaching aids such as videos and images into e-learning platforms. As preparation for students and educators to be ready for the abrupt implementation of e-learning, learning support staff such as IT technicians could help design a step-by-step guide to help with navigating the learning management system, as well as give tips on how to deliver the course content. Moreover, students could form small virtual support groups to assist peers who experience difficulties with the use of e-learning. As far as assessment is concerned, the findings of this study suggest there is a need for educators to be guided on conducting online assessment to match the question structure, marks and time allocated.
